# Abrogating ClC-3 Inhibits LPS-induced Inflammation via Blocking the TLR4/NF-κB Pathway

**DOI:** 10.1038/srep27583

**Published:** 2016-07-01

**Authors:** Nan-lin Xiang, Jun Liu, Yun-jian Liao, You-wei Huang, Zheng Wu, Zhi-quan Bai, Xi Lin, Jian-hua Zhang

**Affiliations:** 1 Department of Pharmacology, Medical College, Jinan University, Guangzhou 510632, China; 2 Department of Physiology, Medical College, Jinan University, Guangzhou 510632, China; 3 Department of Developmental and Regenerative Biology, Jinan University, Guangzhou 510632, China; 4 Department of Key Laboratory for Environmental Exposure and Health, Environment College, Jinan University, Guangzhou 510632, China; 5 Department of Guangzhou Overseas Chinese Hospital, Jinan University, Guangzhou 510632, China; 6 Department of Cardiology, the Sun Yat-sen Memorial Hospital, Sun Yat-sen University, Guangzhou 510120, China

## Abstract

This study investigated the function of a chloride channel blocker, DIDS. Both *in vitro* and *in vivo* studies found that DIDS significantly inhibits lipopolysaccharide (LPS)-induced release of proin flammatory cytokines. Here, we show that DIDS inhibits LPS-induced inflammation, as shown by downregulation of inflammatory cytokines via inhibition of the TLR4/NF-κB pathway. Furthermore, we show that ClC-3siRNA transfection reduces LPS-induced pro-inflammation in Raw264.7 cells, indicating that ClC-3 is involved in the inhibitory effect of DIDS during LPS-induced cytokines release. *In vivo*, DIDS reduced LPS-induced mortality, decreased LPS-induced organic damage, and down-regulated LPS-induced expression of inflammatory cytokines. In sum, we demonstrate that ClC-3 is a pro-inflammatory factor and that inhibition of ClC-3 inhibits inflammatory induction both *in vitro* and *in vivo*, suggesting that ClC-3 is a potential anti-inflammatory target.

Inflammation is an essential immune response that is characterized by pain, swelling, redness, heat, and impaired function^1^. It is a response to traumatic, infectious, post-ischemic, toxic, or autoimmune injury. However, uncontrolled inflammation can lead to disease,and inflammation is now believed to be responsible for several disease conditions^2^. Macrophages are important immune cells in the body, and possess anti-infection, anti-tumor and immune regulatory functions. Macrophages are also central inflammatory mediators.

Macrophages have been at the heart of immune research for over a century and are an integral component of innate immunity. Macrophages are often viewed as terminally differentiated monocytic phagocytes^3^. Macrophages are activated by a variety of inflammatory stimuli, of which bacterial lipopolysaccharide plays an important role. Lipopolysaccharide (LPS), a structural component of the outer membrane of Gram-negative bacteria, is one of the most effective stimulators of the immune system^4^.

4,4′-Diisothiocyanostilbene-2,2′-disulfonic acid (DIDS) is a non-specific and general volume regulated chloride channel blocker. DIDS inhibits cell proliferation and [^3^H]-thymidine incorporation in rat aortic vascular smooth muscle cells (VSMCs) and prevents cell apoptosis in rabbit cardiomyocytes^5^,^6^.

Nuclear factor kappa B (NF-κB), a common transcription factor, regulates various genes encoding inflammatory mediators and acts as an important downstream target of MAPK signaling pathways in inflammatory and immune responses^7^,^8^. NF-κB signaling is a well-studied classical signaling pathway that is involved in the inflammatory response, as well as the regulation of cell proliferation and apoptosis.

Chloride channels play important functional roles in diverse processes, such as blood pressure regulation, cell cycle and apoptosis, muscle tone, volume regulation, synaptic transmission and cellular excitability in mammals^9^. Chloride channels are involved in a diverse set of functions in normal physiology and acquired diseases^10^, ClC-3 is closely related to the inflammatory response. Moreover, ClC-3 is an essential regulator of nuclear factor NF-κB signaling, because ClC-3 deficiency significantly decreases NF-κB activity in mouse aortic smooth muscle cells^11^. In mouse aortic smooth muscle cells, interleukin1 (IL1)-α and tumor necrosis factor (TNF)–α activate a chloride conductance, and this current is dependent on ClC-3 expression^12^.

In our study, we investigated the effect of ClC-3 on LPS-induced inflammatiory cytokines and TLR4/NF-κB pathway activation *in vivo* and *in vitro*. Our results demostrate that ClC-3 inhibits LPS-induced inflammation by inhibiting the TLR4/ NF-κB pathway.

## Materials and Methods

### Cell Line

The mouse macrophage cell line RAW264.7 was obtained from the American Type Culture Collection.Cells were cultured in Dulbecco’s modified Eagle’s medium supplemented with 10% FBS in a humidified incubator containing 5% CO_2_ at 37 °C. The medium was replaced every day and the cells were passed every 2–3 days to maintain logarithmic growth.

### Animals

The Animal Ethical and Welfare Committee of Jinan University approved the animal studies. Mice (6–8 weeks old, half male and half female, weighing 20–24 g) were obtained from the Medical Experimental Animal Center of Guangzhou, Guangdong Province, China. The handling of animals and experimental procedures were conducted in accordance with experimental animal guidelines.

### Reagent

DIDS (Sigma, USA) and LPS (*Escherichia coli*, 0111:B4) were purchased from Sigma and reconstituted in PBS (500 ML, 20012-027, GIBCO).

### Reagent treatment of macrophages *in vitro*

For the western-blotting analysis of inflammatory cytokine activation, 5 × 10^5^ RAW264.7 cells were seeded in 6-well plates for 30 min. Cells were then treated with or without DIDS (100 μmol/L), and then with or without LPS (100 ng/mL) in serum-free DMEM. After 6 h, the cells were lysed in lysis buffer. For the western blotting analysis of NF-κB activation, cells were treated similarly with LPS or DIDS, but after 30 min, they were lysed in lysis buffer containing a protease inhibitor cocktail. For ClC-3 siRNA transfection, 2.5 × 10^5^ RAW264.7 cells were seeded in 6-well plates and transfection was performed according to the manufacturer’s instructions. At 24 hours after transfection, cells were similarly treated with LPS or DIDS, and after 6 h, they were lysed in lysis buffer. For the inflammatory cytokine array, conditioned media following exosome treatment of Raw264.7 cells were concentrated and subsequently applied to the Ray Biotech (Norcross, GA) Mouse Inflammation Antibody Array G series I and processed according to the manufacturer’s instructions.

### Reagent treatment of mice

For the fatality rate analysis of mice, DIDS (14 mg/kg) or normal saline was injected into 6–8 week old mice (half male and half female) by an intraperitoneal route, The mice were pretreated for 30 min, and then injected with LPS (30 mg/kg) or normal saline intraperitoneally. At 12 h, 24 h and 48 h, the fatality rate of the mice in each group was determined. For the hepatorenal function analysis of the mice, the mice were similarly treated with LPS or DIDS. After 4 h, the blood from the mice in each group was collected from the eyes for ELISA and values of ALT and BUN. Then, the mice were sacrificed by cervical vertebra dislocation, and liver and kidney were isolated from the mice in each group and fixed in 4% PFA for HE staining. For the western-blotting analysis of primary macrophages, mice were similarly treated with LPS or DIDS. After 4 h, the mice in each group were sacrificed by cervical vertebra dislocation and intraperitoneally injected aseptically with 10 ml of DMEM. The peritoneal wall was massaged, and ascites were collected and centrifuged at 2000 r/min for 10 min. The supernatant was discarded and cells were washed 2–3 times with DMEM, and then they were seeded in 6-well plates in a humidified incubator containing 5% CO_2_ at 37 °C. After 1 h non-adherent cells were removed and the adherent cells were allowed to grow. These cells were then lysed in lysis buffer.

### Cell transfection

ClC-3 dsRNA was synthesized using a Silencer small interfering RNA (siRNA) construction kit (Ambion, Austin, TX) according to the manufacturer’s instructions. RAW264.7 cells were transfected with dsRNA (10 nM) using Lipofectamine 2000 (Invitrogen) according to the manufacturer’s instructions.

### Western blot analysis

RAW264.7 cells were lysed in RIPA buffer (20 mM Tris, 150 mM NaCl, 1% Nonidet P-40, 0.1 mM EDTA). For the phosphorylation experiment, cells were lysed in RIPA buffer containing a protease inhibitor cocktail (Roche Diagnostics, Indianapolis, IN). We then resolved the proteins by SDS-PAGE under non-reducing conditions and transferred the proteins to PVDF membranes using a semidry transfer apparatus (Bio-Rad). We blocked the membranes with 5% milk for 1 h at room temperature and then incubated them with primary antibodies overnight at 4 °C. After rinsing 3 times, we incubated the immunocomplexes with horseradish peroxidase–conjugated anti-rabbit or anti-mouse IgG (1:2000; Sigma) and visualized the membranes with the Mo-lecular Analyst software (Bio-Rad). The following primary antibodies were used: anti-ClC-3 (1:1,000; ab86192; Abcam), anti-IL1α (1:1,000; 5273; Cell Signaling Technology), anti-IL1β (1:1,000; 12426; Cell Signaling Technology), anti-IL6 (1:1,000; 12912; Cell Signaling Technology), anti-TNFα (1:1,000; 11948S; Cell Signaling Technology) anti-TLR4 (1:1,000; 14358s; Cell Signaling Technology), anti-phospho-p65 (1:1,000; 3033; Cell Signaling Technology), anti-p65 (1:1,000; 8242; Cell Signaling Technology), anti-phospho-IκBα (1:1,000; 9246; Cell Signaling Technology), anti-IκBα (1:1,000; sc-371; Santa Cruz), anti-phospho-JNK (1:500; 9251; Cell Signaling Technology), anti-JNK (1:1,000; 9258; Cell Signaling Technology), anti-phospho-p38 (1:1,000; 9215; Cell Signaling Technology), anti-p38 (1:1,000; 8680; Cell Signaling Technology), anti-phospho-Erk (1:1,000; 4284; Cell Signaling Technology), anti-Erk (1:1,000; 4696; Cell Signaling Technology), anti-phospho-c-jun (3270; 1:1,000; Cell Signaling Technology), anti-c-jun (1:1,000; 9160; Cell Signaling Technology), and anti-β-actin (1:20,000; A1978; Sigma).

### Quantitative Real-Time PCR

Total RNA was extracted from RAW264.7 cells using Trizol Reagent (Invitrogen). cDNA was synthesized from equivalent amounts of RNA with AffinityScript reverse transcriptase and an oligo(dT) primer (Stratagene). Quantitative real-time RT-PCR was performed in an iCycler RT-PCR system (Bio-Rad, Munich, Germany) using the iQ™ SYBR^®^ Green PCR kit (Bio-Rad, Munich, Germany). The primers for ClC-3 are as follows: forward, 5′-CAA UGG AUU UCC UGU CAU ATT-3′ and reverse, 5′-UAU GAC AGG AAA UCC AUU GTA-3.

### ELISA

Serum was collected and analyzed for the production of IL1-α (RayBiotech, Norcross, America), IL1-β (RayBiotech, Norcross, America), IL-6 (RayBiotech, Norcross, America), and TNF-α (RayBiotech, Norcross, America) by using ELISA in accordance with the manufacturer’s instructions.

### Hematoxylin and Eosin Staining

Mice were sacrificed by cervical vertebra dislocation. The kidneys and liver were isolated from mice in each group. Organs were fixed in 4% PFA for 48 h and then routinely processed to paraffin blocks, sectioned at approximately 5 μm. The sections from each organ were routinely stained with H&E staining, and then cover slipped and examined under a light microscope.

### Assay for Serum Alanine Aminotransferase (ALT) and Blood Urea Nitrogen (BUN)

To assay for the serum ALT and BUN levels, blood from mice in each group was collected by sampling from eyeballs. Serum (50 ul) was mixed with 0.5 ml of ALT or BUN assay solution (JianChen, NanJing, China) and then measured in a spectrophotometer following the supplier’s protocol.

### Statistical Analysis

Data were analyzed using independent sample t tests or two-way ANOVA when appropriate. For all quantitative data, statistical analyses were performed using GraphPad software. All data are expressed as the mean ± SEM. Values of P <0.05 were considered significant.

## Results

### Chloride Channel Blocker DIDS Down-regulates LPS-Induced Production of Inflammatory Cytokines in RAW264.7 cells

**Figure 1:**
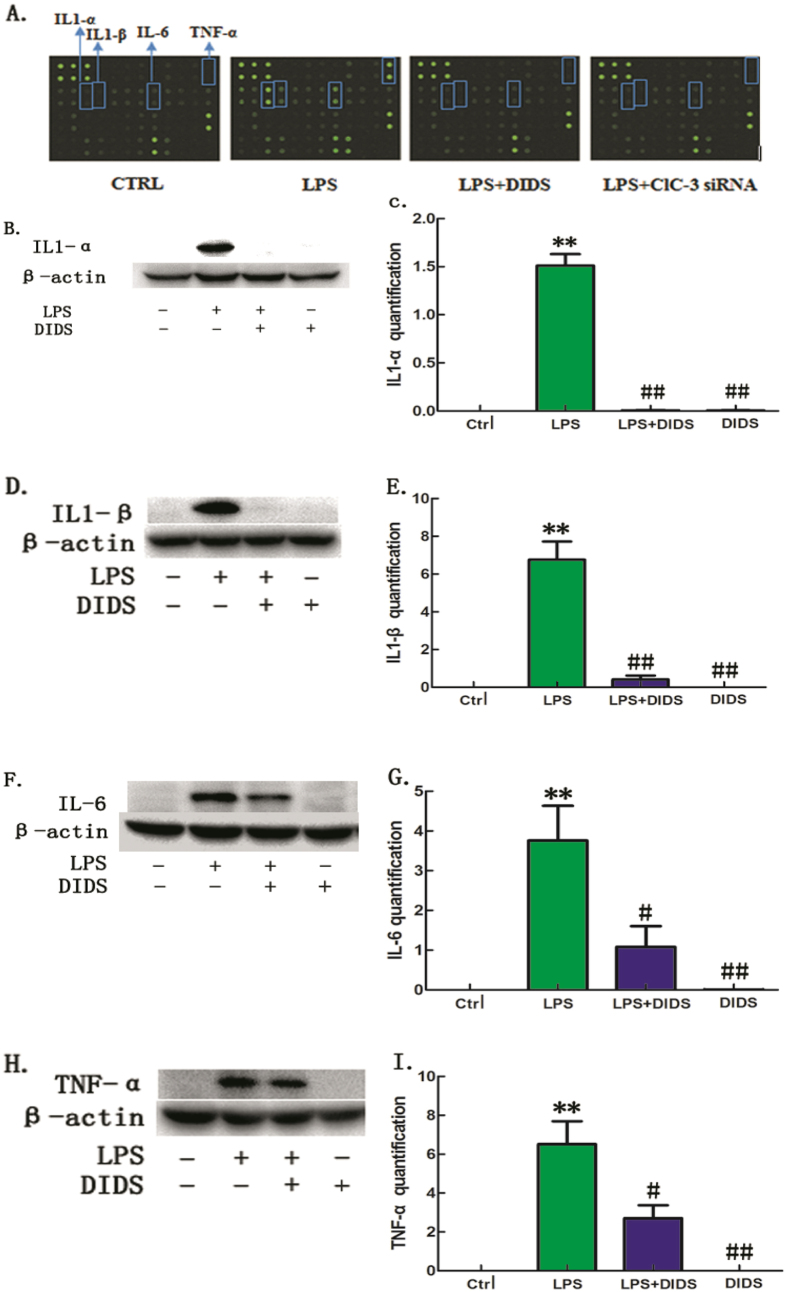
Chloride Channel Blocker DIDS Down-regulates LPS-induced Production of Inflammatory Cytokines in RAW264.7 Cells. (**A**) Inflammatory cytokine array analysis of IL1-α, IL1-β, IL-6 and TNF-α. The expression of these inflammatory cytokines is highly induced by LPS, but down-regulated by DIDS and ClC-3 siRNA. (**B–I**) Western -blotting of IL1-α, IL1-β, IL-6 and TNF-α in RAW264.7 cells in each group. Quantification of western -blotting band intensity of IL1-α, IL1-β, IL-6 and TNF-α corrected for the intensity of β-actin with image-analysis software. (**P < 0.01 vs control, ^#^P < 0.05, ^##^P < 0.01 vs LPS; n = 3).

We used an inflammatory cytokine array to identify cytokines secreted into Raw264.7 cell culture supernatants. Inflammatory production of IL1-α, IL1-β, IL-6 and TNF-α was induced by LPS (100 ng/mL). Interestingly, both DIDS (100 μmol/L) and ClC-3 siRNA significantly down-regulated the production of IL1-α, IL1-β, IL-6 and TNF-α (Fig. 1A). The expression of other inflammatory cytokines in each group are showed in the supplementary material (Fig. S1). The array results were confirmed by western-blotting to assess the role of the mouse macrophage cell line RAW264.7 in responses to LPS stimuli. We treated RAW264.7 with LPS (100 ng/mL) and then determined whether DIDS inhibits the release of inflammatory cytokines. DIDS (100 μmol/L) was used to pretreate cells for 30 min. However, when RAW264.7 cells were incubated with DIDS combined with a stimulatory dose of LPS, IL1-α (Fig. 1B,C), IL1-β (Fig. 1D,E), IL-6 (Fig. 1F,G), and TNF-α (Fig. 1H,I) production were significantly reduced compared to the response to LPS alone. This suggests that DIDS down-regulates the LPS-induced production of inflammatory cytokines in macrophages.

### ClC-3siRNA transfection reduces LPS-induced production of cytokine IL1-α

To observe the distribution of the family of chloride channel proteins ClC-1~ClC-7 in Raw264.7 cells, the mRNA relative expression levels of ClC-1~ClC-7 were assayed by quantitative PCR. The expression of ClC-3 mRNA in RAW264.7 cells was the highest, followed by ClC-6 and ClC-7 (Fig. S2A).

**Figure 2:**
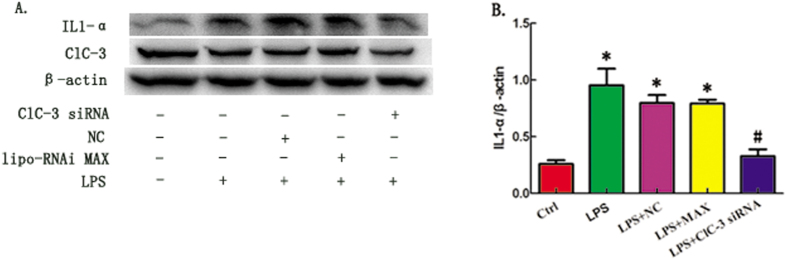
ClC-3siRNA Transfection Reduces LPS-induced Production of Cytokine IL1-α. (**A**) Cell lysates of ClC-3 siRNA and DIDS in each group were prepared and immunoblotted with ClC-3 and IL1-α antibodies. (**B**) Quantification of western-blotting band intensity fpr IL1-α corrected for the intensity of β-actin with image-analysis software. (*P < 0.05 vs control, ^#^P < 0.05 vs LPS; n = 4).

To determine whether ClC-3 is involved in the LPS-induced production of cytokine IL1-α in Raw264.7 cells, we silenced the expression of ClC-3 in RAW264.7 cells by stable transfection of a vector expressing ClC-3 siRNA. ClC-3 expression was significantly down-regulated by ClC-3 siRNA, but not by the corresponding scrambled controls (Fig. S2B). We found that the production of IL1-α in RAW264.7 cells upon LPS stimulation was significantly inhibited after ClC-3 silencing (Fig. 2A,B). This suggests that the chloride channel protein ClC-3 is involved in the LPS-induced production of cytokine IL1-α in Raw264.7 cells.

### Chloride Channel Blocker DIDS and Transfected ClC-3siRNA Reduced LPS-Induced Expression of TLR4 in Macrophages

**Figure 3:**
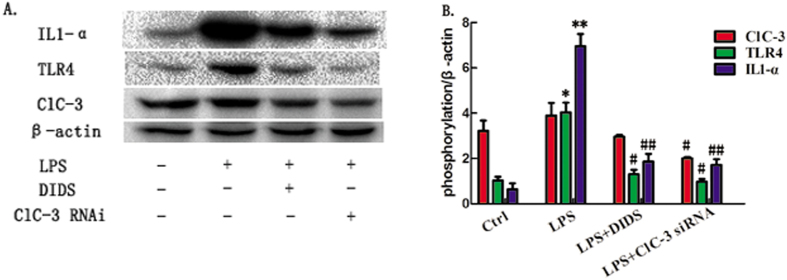
Chloride Channel Blocker DIDS and ClC-3 siRNA Transfection Reduces LPS-Induced Expression of TLR4 in Macrophages. (**A**) Cell lysates of ClC-3 siRNA and DIDS in each group were prepared and immunoblotted with ClC-3, TLR4 and IL1-α antibodies. (**B**) Quantification of western -blotting band intensity of IL1-α and TLR4, respectively, corrected for the intensity of β-actin with image-analysis software. (*P < 0.05, **P < 0.01 vs control, ^#^P < 0.05, ^##^P < 0.01 vs LPS; n = 3).

LPS stimulates TLR4 signaling, leading to proinflammatory cytokine release^13^. Therefore, to determine whether ClC-3 is involved in the regulation of TLR4 signaling, we silenced the expression of ClC-3 in RAW264.7 cells by stablely transfecting a vector expressing ClC-3 siRNA. ClC-3 expression was clearly down-regulated by ClC-3 siRNA, but not by the corresponding scrambled controls. We used western blotting to identify the isoform of TLR4 in RAW264.7 cells induced by LPS stimulation after ClC-3 silencing. When ClC-3 was silenced, the expression of LPS-induced TLR4 in Raw264.7 cells was down-regulated (Fig. 3A,B), suggesting that TLR4 is involved in blocking LPS-induced inflammation. Therefore, DIDS likely inhibits LPS-induced inflammatory cytokines via TLR4 in RAW264.7 cells.

### Chloride channel blocker DIDS and ClC-3siRNA transfection inhibit LPS-induced production of inflammatory cytokines via the NF-κB signaling pathway

**Figure 4:**
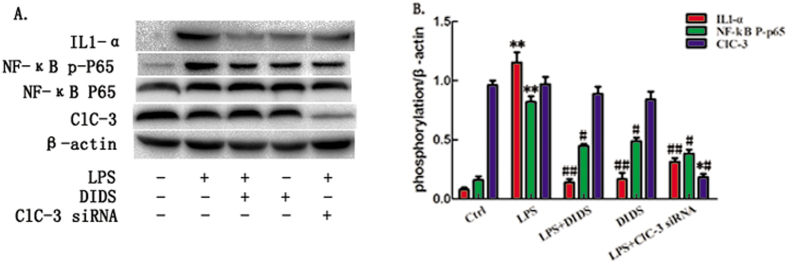
Chloride Channel Blocker DIDS and ClC-3 siRNA transfection Inhibit LPS-induced Production of Inflammatory Cytokines via NF-κB Signaling Pathway. (**A**) RAW264.7 cells were pretreated with either DIDS or ClC-3siRNA, followed by stimulation with LPS for the indicated time points. NF-κB phosphorylation was determined by Western -blotting. (**B**) Quantification of western -blotting band intensity of IL1-α and p-p65, corrected for the intensity of β-actin with image-analysis software. (**P < 0.01 vs control, ^#^P < 0.05, ^##^P < 0.01 vs LPS; n = 3).

To determine which signaling pathway intersects the TLR signaling pathway, we examined the kinetics of the activation of three MAPKs and the NF-kB pathway, which are all downstream of TLR stimulation^14^. Treatment with LPS induced phosphorylation in the MAPK pathways in RAW264.7 cells, including NF-kB (Fig. 4A,B), C-jun (Fig. S3A,B), ERK1/2 (Fig. S3C,D) and P38MAPK (Fig. S3E,F). The phosphorylation of NF-kB P65 was significantly attenuated by DIDS pretreatment, followed by a decrease of IL1-α. By contrast, DIDS pretreatment did not affect the phosphorylation of the C-jun, ERK1/2 and P38 MAPK pathways. Therefore, the principal effect in TLR downstream signaling that is involved in DIDS inhibition of LPS-induced inflammatory cytokine production is the NF-κB signaling pathway. We conclude that down-regulation of ClC-3 inhibits LPS-induced inflammatory cytokines via the NF-κB signaling pathway.

### Chloride Channel Blocker DIDS Reduces LPS-Induced Mortality in Mice

**Figure 5:**
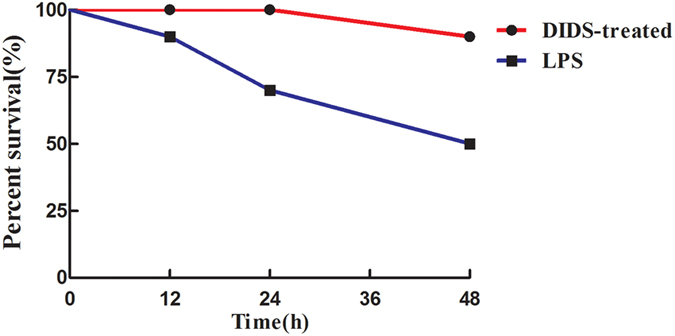
Chloride Channel Blocker DIDS Reduces LPS-Induced Mortality in Mice. DIDS was injected i.p. 30 min before coinjection with LPS. The survival rate was measured after 12 h, 24 h and 48 h.

To determine whether the chloride channel blocker DIDS reduces mortality by decreasing the inflammatory response in mice, we pretreated mice with DIDS (14 mg/kg) 30 min before LPS (30 mg/kg) injection. The survival rate of mice at 12 h, 24 h and 48 h after injection with LPS was 90%, 70%, and 50%, respectively. DIDS-infused mice survived at 12 h and 24 h, and one mouse died at 48 h (Fig. 5), which indicates the protective role of DIDS. This difference was also observed for the weight, in that the weight of LPS-injected mice was significantly decreased (data not shown). LPS-infused mice exhibited convulsions, arcus dorsalis and tremble, but DIDS-treated mice did not show any of these behaviors. These data indicate that DIDS reduces LPS-induced mortality in mice.

### Chloride Channel Blocker DIDS Reduces LPS-Induced Organ Damage in Mice

**Figure 6:**
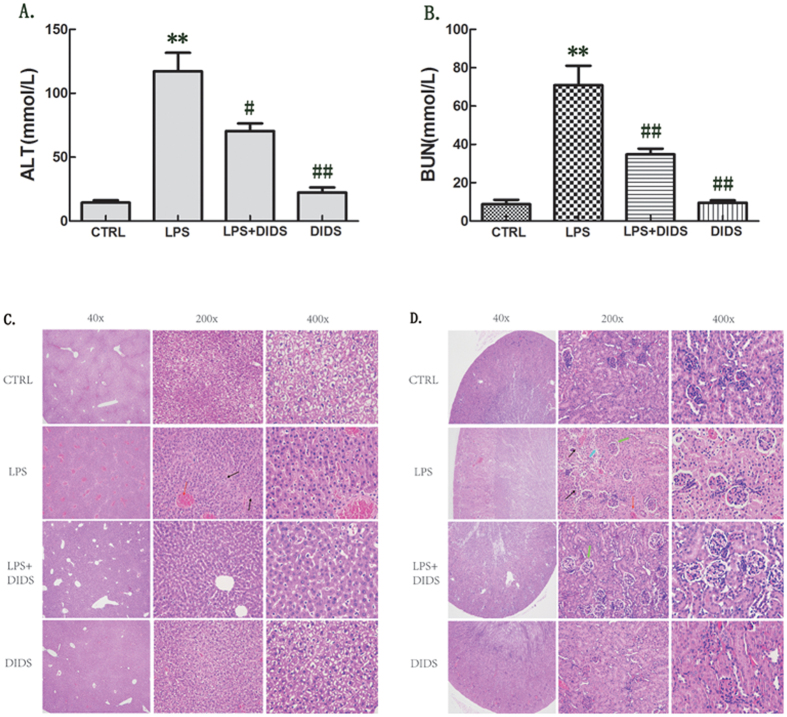
Chloride Channel Blocker DIDS Reduces LPS-induced Organ Damage in Mice. (**A,B**) DIDS was i.p.-injected into mice 6 h before LPS injection. Serum samples were collected 6 h after coinjection, and ALT and BUN activity in the serum were determined. (**C,D**) H&E staining of hepatorenal tissue from each group of mice at different magnifications. Livers of LPS-treated mice (Fig. 5C). As shown by the black arrow, the topical area of the liver tissue shows many fat particles. As shown by the red arrow, the hepatic veins are filled with red blood cells. However, in the livers of DIDS/LPS-treated mice, a small amount of hepatic sinus is slightly dilated and liver damage is decreased. The same expression is observed in the kidney tissue, of LPS-infused mice (Fig. 5D). As shown by the black arrow, there is a decrease in the number of glomerular capillary loop, and glomerular atrophy. As shown by the green arrow, there is topical proliferation of glomerular epithelial cells. As shown by the blue arrow, the nucleus of renal tubular epithelial cells are condensed, undergoing necrocytosis, and there is degradation of the cytoplasm protein and the structure of the kidney tubules has disappeared. As shown by the red arrow, there is dilatation and hyperemia of the blood capillary in the renal tubulointerstitial space. In the kidney tissue of DIDS/LPS-treated mice, the structure of the glomerulus and kidney tubules are integrated, with only an small amount of topical proliferation in glomerular epithelial cells, as the green arrow shown. (**P < 0.01 vs control, ^#^P < 0.05, ^##^P < 0.01 vs LPS; n = 5).

In our previous studies, we showed that DIDS significantly inhibits LPS-induced inflammatory cytokines *in vitro*. We therefore determined whether the *in vivo* results are consistent with our previous studies. We injected mice intraperitoneally (i.p.) with LPS (30 mg/kg), and the dose of LPS was selected to induce acute inflammation. In addition, the dose of DIDS (14 mg/kg) was selected because it did not induce any side effects and maintained drug action. ALT and BUN were tested in liver and kidney as functional indicators, respectively. The protective effect of DIDS was confirmed by the results the revealed that DIDS/LPS-treated mice had a lower elevation of serum ALT and BUN levels compared to LPS-infused mice (Fig. 6A,B). Furthermore, pathology analysis showed that LPS-infused hepatorenal tissue produced a large number of inflammatory cytokines and had severe tissue damage. DIDS-treated mice had reduced LPS-infused hepatorenal destruction and inflammatory cell infiltration (Fig. 6C,D). These data indicates that DIDS improves organ function and reduces organ damage in LPS-induced mice.

### Chloride Channel Blocker DIDS Down-regulates LPS-Induced Expression of Inflammatory Cytokines in Mice

**Figure 7:**
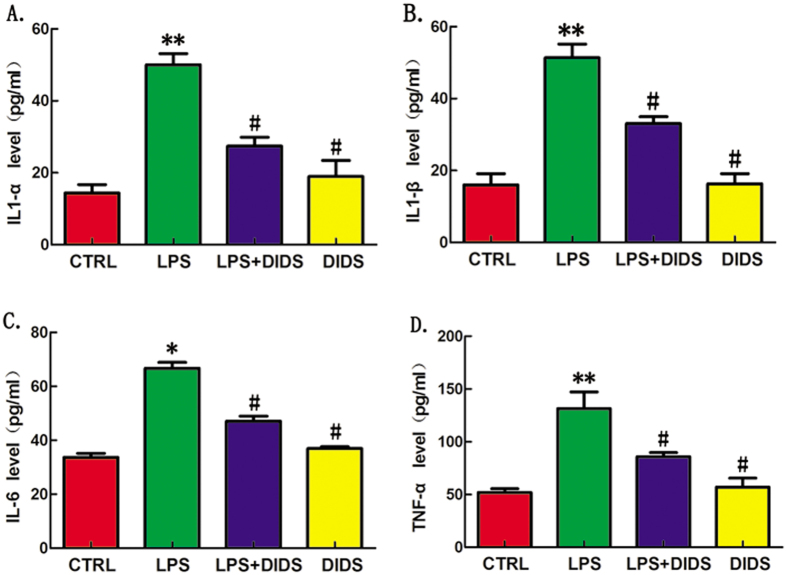
Chloride Channel Blocker DIDS Down-regulates LPS-induced Expression of Inflammatory Cytokines in Mice. DIDS was injected i.p. 30 min before coinjection with LPS. (**A**) Serum samples in each group were collected at 6 h and IL1-α was measured by ELISA. (**B**) IL1-β was measured by ELISA. (**C**) IL-6 was measured by ELISA. (**D**) TNF-α was measured by ELISA. (*P < 0.05, **P < 0.01 vs control, ^#^P < 0.05 vs LPS; n = 3).

Overproduction of IL1-α, IL1-β and TNF-α are major factors involved in the lethal effect of LPS^15^. To determine whether this is due to elevated TNF-α production, mice were injected intraperitoneally (i.p.) with LPS (30 mg/kg) in the presence or absence of DIDS (14 mg/kg). After 4 h, mice were sacrificed, and plasma IL1-α (Fig. 7A), IL1-β (Fig. 7B), IL-6 (Fig. 7C) and TNF-α (Fig. 7D) were measured. We found that cytokines were down-regulated upon administration of DIDS, compared to the injection of LPS alone. The data show that DIDS inhibits LPS-induced secretion of inflammatory cytokines in mice.

### Chloride Channel Blocker DIDS Down-regulates LPS-Induced Production of IL1-α and NF-κB phosphorylated P65 in Primary Macrophages

**Figure 8:**
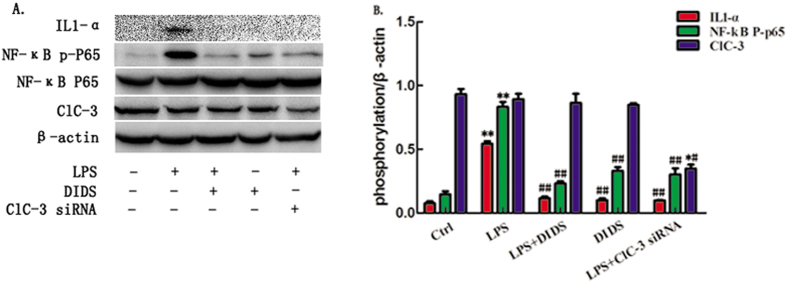
Chloride Channel Blocker DIDS Down-regulates LPS-Induced Production of IL1-α and NF-κB Phosphorylated P65 in Primary Macrophages. (**A**) Western -blotting of IL1-α and NF-κB phosphorylated P65 in primary macrophages. (**B**) Quantification of western-blotting band intensity of IL-1α and p-p65, corrected for the intensity of β-actin with image-analysis software. (**P < 0.01 vs control, ^#^P < 0.05, ^##^<0.01 vs LPS; n = 3).

To further verify chloride channel involvement in LPS-induced inflammation *in vivo*, primary macrophages were separated and cultured from extracted ascites from the peritoneal cavity of mice in each group. The expression of IL1-α and NF-κB phosphorylated P65 were analyzsed by western-blotting in primary macrophages (Fig. 8). The data indicate that DIDS inhibits LPS-induced production of IL1-α and NF-κB phosphorylated P65 in primary macrophages.

## Discussion

Acute inflammation plays an important role in the pathophysiology of systemic inflammatory response syndrome (SIRS), a lethal inflammatory disease caused by trauma, burns, and infection^16^. Dysregulation of host responses to LPS can lead to systemic inflammatory conditions, such as sepsis^17^. The pathophysiology of inflammation is complicated. At present, there has been little reported research regarding the mechanism of action and correlation between chloride channels and inflammation. In this study, we found that chloride channel blocker DIDS and a decrease of ClC-3 downregulated LPS-induced inflammatory cytokine IL1-α *in vivo* and *in vitro*. Furthermore, DIDS and a decrease of ClC-3 inhibited the LPS-induced inflammatory response via the TLR4/NF-κB pathway. In conclusion, the chloride channel ClC-3 palys an important role in the inhibition of inflammation.

Lipopolysaccharide (LPS) acts on cell-membrane receptors, which change gene expression through intracellular signaling cascades. LPS-induced systemic inflammation or endotoxemia is a well-accepted and validated model of systemic inflammatory response syndrome characterized by pathological changes that strongly resemble sepsis^18^,^19^. LPS mediates the activation of endothelial cells, smooth muscle cells, fibroblasts, epithelial cells and monocytes,and induces inflammatory cytokines, chemokines, growth factors and other factors, such as interleukins and tumor necrosis factor^20^.

Recent studies found that chloride channels have several functions, including the regulation of cell volume, stabilizing membrane potential, signal transduction and transepithelial transport^21^. Chloride may be a type of second messenger in cells: its concentration is regulated by channels and transporters, because it is intracellular concentration is dynamic on both short and long time scales, it is transported into and out of intracellular organelles,and it binds to and regulates the function of a variety of proteins^22^. ClC-3 is a volume-activated chloride channel protein and has the largest distribution in RAW264.7 cells. In this study, we found that ClC-3 is a pro-inflammatory factor,and that specifically targeting CIC-3 with siRNA abrogates the expression of ClC-3 in membranes, further inhibiting inflammation. DIDS is a non-specific blocking agent for chloride channels and is commonly used in volume-activated chloride channels, but the anti-inflammatory mechanism of DIDS is not yet clear. SophiaF^23^ reported that DIDS blocked the hypotonicity-induced inward current and outward current in a reversible manner. Han X^24^ found that DIDS inhibits the arrhythmogenic transient inward current in rabbit cardiac Purkinje fibers. In this study, we hypothesized that DIDS blocked the inward current more effectively than the outward current, thereby inhibiting the expression of the chloride channel protein ClC-3, which is consistent with the results of ClC-3 siRNA transfection.

The toll-like receptors (TLRs) family is one of the best characterized of several cellular effectors for pathogen detection^25^. TLRs are a family of trans-membrane proteins that are widely expressed by eukaryotic cells that recognize ligands present in virtually all types of microorganisms. After binding occurs, the activation of signaling pathways downstream of TLRs plays a major role in directing both innate and adaptive host immune responses^26^. TLR4 is activated by LPS. Then, a multimolecular complex triggers a signaling cascade leading to early phase activation of NF-κB, and also controls the production of pro-inflammatory cytokines, such as IL1-α and TNF-α^27^. TLR4/NF-κB is a classic signaling pathway of inflammation. The increased expression of TLR4 leads to the activation of NF-κB, which is a typical downstream target of TLR4^28^. ClC-3 is an essential regulator of TLR4/NF-κB signaling. For examples ClC-3 deficiency significantly decreases NF-κB activity in mouse aortic smooth muscle cells, and a decrease of the intracellular chloride concentration promotes endothelial cell inflammation by activating the NF-κB pathway^29^.

IL-1 is a master cytokine of local and systemic inflammation^30^. In this study, we found that knockdown of ClC-3 in RAW264.7 cells downregulates the membrane receptor TLR4 and cytokine IL1-α, which indicates that ClC-3 is highly involved in the TLR4/NF-κB pathway and subsequent expression of IL1-α in RAW264.7 cells.

Furthermore, to clarify whether chloride channels have anti-inflammatory effects *in vivo*, we selected a mouse model of sepsis, characterized as systemic organic damage induced by a severe inflammatory reaction. DIDS reduces the LPS-induced mortality of sepsis. Moreover, we also observed that DIDS inhibits the expression and secretion of serum IL1-α production, improves organic function, reduces organic damage and inhibits the activation of macrophages, which indicates that DIDS inhibits the development of an inflammatory response by inhibiting the expression of IL1-α.

## Conclusions

In conclusion, our study shows the effect of ClC-3 on LPS-induced inflammatory cytokines and TLR4/NF-κB pathway activation. The present study demonstrates that ClC-3 inhibits the LPS-induced inflammatory response by inhibiting the TLR4/ NF-κB pathway *in vivo* and *in vitro*. These results provide a new view on the inhibition of inflammation based on chloride channels, and lay the foundation for clinical research into chloride channels in the future.

## Additional Information

**How to cite this article**: Xiang, N.-l. *et al.* Abrogating ClC-3 Inhibits LPS-induced Inflammation via Blocking the TLR4/NF-κB Pathway. *Sci. Rep.* **6**, 27583; doi: 10.1038/srep27583 (2016).

## Supplementary Material

Supplementary Figure S1

Supplementary Figure S2

Supplementary Figure S3
