# Supplementary material for: Abrogating ClC-3 Inhibits LPS-induced Inflammation via Blocking the TLR4/NF-κB Pathway
Source: Sci Rep. 2016 Jul 1;6:27583. doi: 10.1038/srep27583 (PMC4929440; doi:10.1038/srep27583)
Supplement: Supplementary Figure S1 [file srep27583-s1.pdf]

Abrogating ClC-3 Inhibits LPS-Induced Inflammation via Blocking TLR4/ NF-κB Pathway

Nan-lin Xiang[1#], Jun Liu[2#], Yun-jian Liao[1], You-wei Huang[1], Zheng Wu[4], Zhi-quan Bai[2], Xi Lin[1,3*], Jian-hua Zhang [5,6*]

[1]Department of Pharmacology, Medical College, Jinan University, Guangzhou 510632, China

[2]Department of Physiology, Medical College, Jinan University, Guangzhou 510632, China

[3]Department of Key Laboratory for Environmental Exposure and Health, Environment College, Jinan University, Guangzhou 510632, China

[4]Department of Developmental and Regenerative Biology, Jinan University, Guangzhou 510632, China

[5]Department of Guangzhou Overseas Chinese Hospital, Jinan University , Guangzhou 510632, China

[6]Department of Cardiology, the Sun Yat-sen Memorial Hospital, Sun Yat-sen University, Guangzhou 510120, China

# These two authors contributed equally to this article.

* To whom correspondence should be addressed. E-mail: Linx_jnu@163.com, fax:+86 2085228865

Protein name	Contrl	LPS	LPS+DIDS	LPS+ClC-3 siRNA	LPS/Contrl	LPS+DIDS/Contrl	LPS+ClC-3 siRNA/Contrl
POS	13,294	13,294	13,294	13,294	1.00	1.00	1.00
BLC	29	68	39	33	2.35	1.34	1.12
CD30LG	50	48	57	49	0.97	1.14	1.00
CCL11	13	22	12	10	1.75	0.94	0.77
CCL24	107	271	121	144	2.53	1.13	1.35
FASLG	14	16	21	22	1.19	1.55	1.62
CX3CL1	47	108	38	55	2.31	0.81	1.18
CSF3	141	201	139	133	1.43	0.99	0.94
CSF2	175	419	141	164	2.40	0.81	0.94
INF-γ	211	260	193	215	1.23	0.91	1.02
IL-1α	41	10,288	72	85	254.02	1.77	2.11
IL-1β	20	417	28	22	21.40	1.44	1.12
IL-2	166	223	153	165	1.34	0.92	0.99
IL-3	157	207	156	159	1.32	0.99	1.01
IL-4	180	219	188	179	1.22	1.04	1.00
IL-6	133	724	123	140	5.47	0.93	1.05
IL-9	170	236	153	171	1.39	0.90	1.01
IL-10	105	155	107	104	1.47	1.02	0.99
IL-12 p40/p70	35	45	29	33	1.30	0.84	0.96
IL-12 p70	173	224	158	157	1.30	0.92	0.91
IL-13	15	22	24	22	1.54	1.69	1.51
IL-17A	246	273	230	241	1.11	0.94	0.98
I-TAC	16	25	17	21	1.56	1.05	1.30
KC	18	12	16	17	0.68	0.90	0.99
Leptin	16	16	15	14	1.04	0.99	0.92
LIX	47	82	48	51	1.75	1.03	1.09
XCL1	111	125	124	117	1.13	1.12	1.05
MCP1	163	203	144	170	1.25	0.88	1.04
CSF1	24	28	20	22	1.17	0.87	0.95
MIG	6	20	9	11	3.29	1.44	1.86
MIP-1α	10	48	21	37	5.03	2.20	3.85
MIP-1γ	15,555	20,916	19,080	18,706	1.34	1.23	1.20
RANTES	16	78	18	21	5.03	1.18	1.34
SDF-1	23	32	26	34	1.43	1.15	1.49
TCA-3	262	525	229	231	2.01	0.88	0.88
TECK	122	362	140	144	2.98	1.15	1.18
TIMP1	18	32	19	12	1.81	1.11	0.70
TIMP2	33	39	43	36	1.20	1.30	1.09
TNF-α	3	145	28	6	57.95	11.21	2.44
sTNFR1	3,206	1,272	2,629	1,687	0.40	0.82	0.53
sTNFR2	278	1,189	328	401	4.28	1.18	1.45

Fig.S1. Mouse Inflammation Antibody Array identifies inflammatory cytokines in RAW264.7 cells.
