# Supplementary material for: Abrogating ClC-3 Inhibits LPS-induced Inflammation via Blocking the TLR4/NF-κB Pathway
Source: Sci Rep. 2016 Jul 1;6:27583. doi: 10.1038/srep27583 (PMC4929440; doi:10.1038/srep27583)
Supplement: Supplementary Figure S3 [file srep27583-s3.pdf]

Abrogating ClC-3 Inhibits LPS-Induced Inflammation via Blocking TLR4/ NF-κB Pathway

Nan-lin Xiang[1#], Jun Liu[2#], Yun-jian Liao[1],You-wei Huang[1], Zheng Wu[4], Zhi-quan Bai[2], Xi Lin[1,3*], Jian-hua Zhang [5,6*]

[1]Department of Pharmacology, Medical College, Jinan University, Guangzhou 510632, China

[2]Department of Physiology, Medical College, Jinan University, Guangzhou 510632, China

[3]Department of Key Laboratory for Environmental Exposure and Health, Environment College, Jinan University, Guangzhou 510632, China

[4]Department of Developmental and Regenerative Biology, Jinan University, Guangzhou 510632, China

[5]Department of Guangzhou Overseas Chinese Hospital, Jinan University , Guangzhou 510632, China

[6]Department of Cardiology, the Sun Yat-sen Memorial Hospital, Sun Yat-sen University, Guangzhou 510120, China

# These two authors contributed equally to this article.

* To whom correspondence should be addressed. E-mail: Linx_jnu@163.com, fax:+86 2085228865

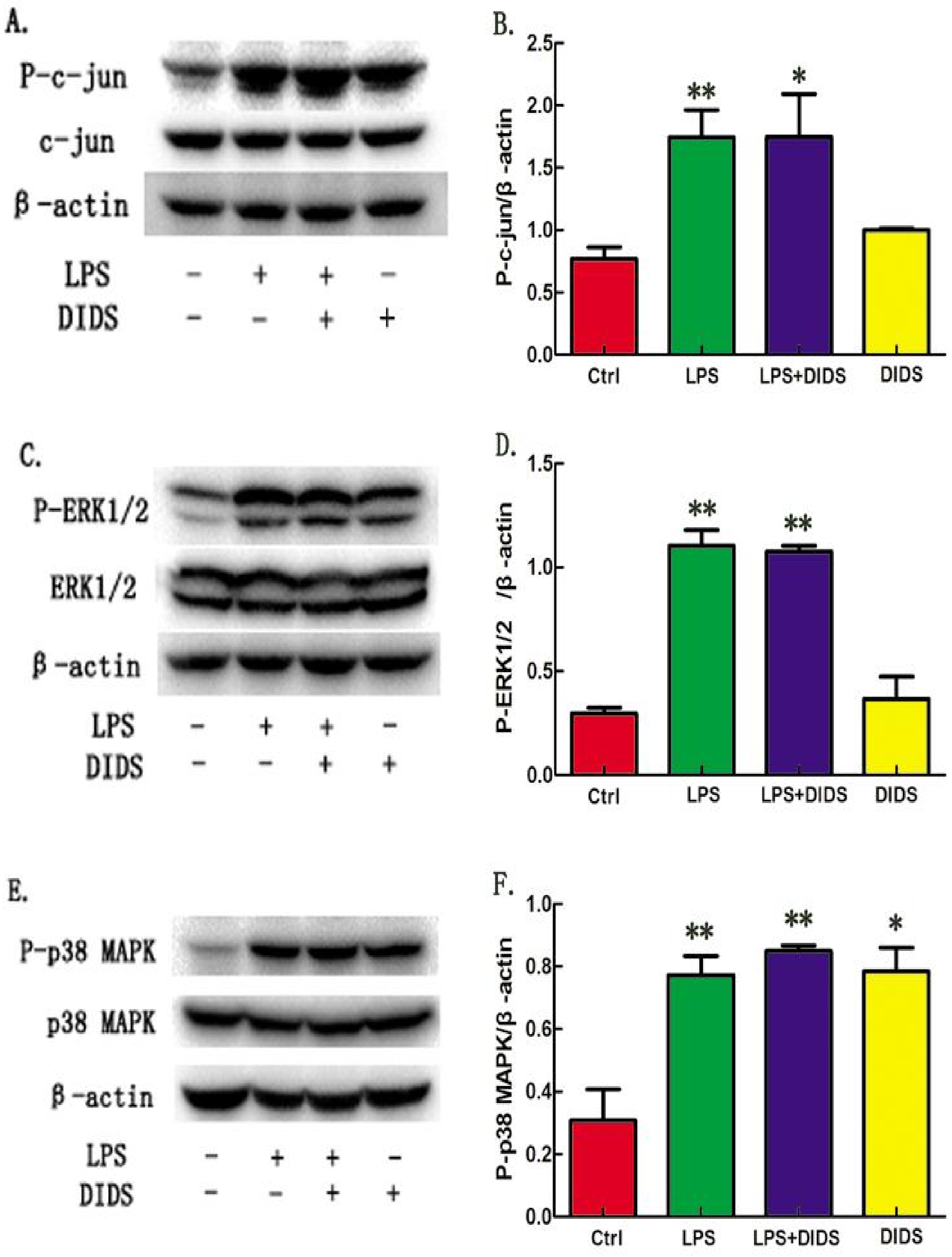

Fig.S3.(A)C-jun,downsteam of JNK signaling pathway, the phosphorylation was determined by Western blot. Data represent mean±SD of three independent experiments. (C) ERK1/2 phosphorylation was determined by Western blot. (E) p38MAPK phosphorylation was determined by Western blot. (B,D,F)Quantification of western blot band intensity of p-c-jun,p-ERK1/2 and p-p38 MAPK, respectively,corrected for the intensity of β-actin with image-analysis software.（$^*P<0.05$,$^{**}P<0.01$ vs control,n=3）
